# Effects of Alcoholic Extracts of Bangladeshi Mangrove *Acanthus ilicifolius* Linn. (Acanthaceae) Leaf and Stem on Atherogenic Model of Wistar Albino Rats

**DOI:** 10.1155/2021/7539037

**Published:** 2021-05-30

**Authors:** Rubaba Karim, Mst Marium Begum, Md. Abdul Alim, Md. Sahab Uddin, Md. Tanvir Kabir, Ashfia Fatima Khan, Tanjina Islam, Shafiul Islam Khan, Md. Sohanur Rahman

**Affiliations:** ^1^Department of Pharmacy, Primeasia University, Dhaka 1213, Bangladesh; ^2^Department of Pharmacy, East West University, Dhaka, Bangladesh; ^3^Department of Chemistry, Bangabandhu Sheikh Mujibur Rahman Science and Technology University, Gopalganj, Bangladesh; ^4^Graduate School of Innovative Life Science, Faculty of Engineering, University of Toyama, Toyama, Japan; ^5^Department of Pharmacy, Southeast University, Dhaka, Bangladesh; ^6^Pharmakon Neuroscience Research Network, Dhaka, Bangladesh; ^7^Department of Pharmacy, Brac University, Dhaka 1212, Bangladesh; ^8^Department of Biochemistry and Molecular Biology, Trust University, Barishal, Ruiya, Nobogram Road, Barishal 8200, Bangladesh

## Abstract

*Acanthus ilicifolius* Linn. (Acanthaceae) is a popular mangrove ethnomedicinal plant that cures several ailments, including asthma, diabetes, cancer, and many others. Our experiment was aimed at evaluating the anti-atherogenic effect of *A. ilicifolius* (leaf and stem) on a high-fat diet-induced atherogenic rat model. Atherosclerosis was developed in 12 weeks. Treatment with the standard drug (3 mg/kg b.w./day, p.o. of Simvastatin), separate doses of methanolic and ethanolic extracts of *A. ilicifolius* leaf (250 and 500 mg/kg b.w./day, p.o.), and stem (200 and 400 mg/kg b.w./day, p.o.) was subsequently conducted for additional 15 days. The anti-atherogenic effect was evaluated by estimating the change in body weight, systolic blood pressure, and lipid profile. Histopathology of aorta, liver, and kidney of atherogenic models was done for further evaluation. The antioxidant effect of different extracts was performed via DPPH (2,2-diphenyl-1-picrylhydrazyl) assay using ascorbic acid as standard. The anticoagulant effect was determined after 15 days of treatment with the same doses of the plant extracts and the standard Warfarin (2 mg/kg b.w./day, p.o.). When compared with atherogenic control, treatment with *A. ilicifolius* significantly reduced (*p* < 0.01) body weight, systolic blood pressure, and serum lipid levels while it elevated HDL (high-density lipoprotein) level in a dose-dependent manner. Moreover, bleeding and clotting time was significantly decreased (*p* < 0.01) under the treatment of plant extracts. The histopathological data showed considerable improvement in tissue morphology after treatment. Our study evidenced that the alcoholic extracts of *A. ilicifolius* leaf and stem have anti-atherogenic properties and may be recommended as a potential herbal remedy for preventing cardiovascular diseases.

## 1. Introduction

Mangrove plants are considered promising sources of phytochemical constituents that are immensely used in ethnopharmaceutical practices worldwide [1]. The secondary metabolites produced by these plants have been proved to possess certain medicinal values. Health practitioners of many developing countries traditionally employ them to cure a wide variety of ailments since ancient times [2, 3]. *A. ilicifolius*, which is indigenously identified as “Hargoza,” is one of the well-known mangrove plants in India, and other tropical areas of Asia, Africa, and northern Australia. In Bangladesh, this plant is wide-spectrum available in large areas of mangrove forest in Sunderbans and the coastal regions of southern districts [4]. *A. ilicifolius* possesses many pharmacological activities, including anti-inflammatory, antioxidant, anti-cancer [5, 6], hepatoprotective, anti-ulcer, anti-osteoporotic [7], antileishmanial, and antimicrobial activities [3, 8], for which it has been used in traditional Indian (Ayurveda) as well as Chinese medicine for many years [3, 9].

Atherosclerosis has markedly gained global attention for decades. It is an inflammatory disease involving several factors that generate lipid deposition in the arterial walls leading to arterial lesions. These factors are considerably total cholesterol, low-density lipoprotein cholesterol, triglycerides, insulin resistance, inflammation, reactive oxygen species or free radicals, and diet containing a large amount of saturated fat [10–16]. Diets rich in lipids have been often applied to develop an atherogenic model using mice or related rodents [16]. Hence, atherogenic animal model could be a promising kit to perceive pathology of the disease and assess the consequence of the treatment pattern.

According to an earlier report, methanolic extract of *A. ilicifolius* exhibited anti-inflammatory activity against rat paw edema [3]. Another investigation indicated that the plant confirmed the anti-diabetic effect in rats and lowered blood glucose levels similar to the standard hypoglycemic drug (glibenclamide) [17]. Leaves and flower extracts of the plant displayed significant antioxidant properties in the past studies [18, 19]. However, the stem extract of *A. ilicifolius* demonstrated potential absorption of free radicals that corresponded to ascorbic acid action [20]. Aqueous extract of roots of this plant exhibited potent anticoagulant and anti-cancer activities in the recent past year [21].

Many phenolic compounds, mainly phenolic acids and phenolic glycosides [22–25], were found in various parts of *A. ilicifolius* that associated with potent antioxidant, anti-cancer, anti-atherosclerotic, antibacterial, antiviral, and anti-inflammatory activities [26–29]. The plant also possessed other significant bioactive compounds, including flavonoids, lignans, phenyl ethanols, and triterpenoids [3], which functioned as antioxidants and were responsible for free radical scavenging properties [30–32].

Concerning the above facts and features about *A. ilicifolius*, the current work aimed to investigate the presence of primary phytoconstituents, the anti-atherosclerotic, and anticoagulant properties of alcoholic extracts of leaves and stem of Bangladeshi mangrove variety of this plant using high-fat diet-induced atherosclerosis in rats. The study further continued to determine antioxidant activities of this plant in order to scientifically support its utilization as a traditional medicine against cardiac diseases.

## 2. Materials and Methods

### 2.1. Drugs, Chemicals, and Nutritional Supplements

Warfarin, Simvastatin, and ascorbic acid were obtained from Eskayef Bangladesh Limited. DPPH (2, 2-diphenyl-1-picrylhydrazyl) and cholesterol were obtained from Sigma Aldrich Chemicals, Germany. Soybean oil and corn starch were purchased from the local market (Unilever food, Bangladesh). The minerals and vitamins for the animal diet were obtained from Merck (Darmstadt, Germany). Lipid profile and blood coagulation test kits (for Activated Partial Thromboplastin Time and Prothrombin Time assays) were procured from Sigma Aldrich Chemicals, Germany. The rest of the chemicals and reagents were purchased from Merck (Darmstadt, Germany), and all were analytical grade chemicals.

### 2.2. Collection and Authentication of Plant

Fresh plant parts (leaves and stems) were collected from St. Martin Island, Bangladesh, in October 2018. The plant parts were identified and certified by a taxonomist of Bangladesh National Herbarium, Dhaka, Bangladesh. A voucher specimen with the accession number DACB 46483 was deposited for further acknowledgment.

### 2.3. Extraction of Plant Parts

The leaves and stem were washed and then shaded dried for consecutive 5 days. The dried leaves and stems were subjected separately to a mechanical grinder to prepare coarse powder. 200 gm of coarse powder of leaves and stems was stored in separate air-tight containers and kept in a dry, cool, and dark place until used for the experiment. The crude extract was prepared by following the previous method with a slight modification [33]. The extraction was done by maceration process using 95%, 400 mL of methanol and ethanol as solvents into which 200 gm of dried powder of leaves and stems soaked separately. The mixtures were kept for 21 days within 22–25°C with infrequent shaking. Later, Whatman filter paper No. 1 was used to filter the extracts, followed by concentrating the filtrates using a rotary evaporator at 45°C (pressure 57 mmHg). The samples were kept unlidded for some days to volatilize the residual solvents. Finally, the residues of methanolic and ethanolic extracts of leaves and stems were collected separately, and the mass of each extract was calculated in percentage according to the following equation:

(1) $$\begin{array}{c} \text{yield }\left(\text{\%}\right)=\frac{\text{mass of crude extract}\times 100}{\text{total mass of dry powder}}. \end{array}$$

The percentage extraction of AILE and AILM was found to be 15% and 23%, respectively, while AISM and AISE were observed to be 17% and 20%.

### 2.4. Screening of Biologically Active Compounds

Freshly prepared extracts were subjected to preliminary phytochemical analysis to identify secondary metabolites like carbohydrates, triterpenoids, alkaloids, glycosides, tannins, flavonoids, and essential oils. Phytochemical tests using standard protocols [33–35] were performed to construct the presence of bioactive compounds in different extracts of *A. ilicifolius*.

### 2.5. Quantitative Analysis of In Vitro Antioxidant Activity

The free radical scavenging properties of different extracts of *A. ilicifolius* were determined against DPPH (2,2-diphenyl-1-picrylhydrazyl) by using the previous method with slight modification [36] where ascorbic acid was considered as the standard. The IC_50_ (50% inhibitory concentration) values of the DPPH assay were determined by a linear regression curve of % inhibition versus sample concentration. Different concentrations of ascorbic acid and plant extracts (1.25, 2.5, 5, 10, 50, and 100 *µ*g/mL) have been prepared. 100 *µ*L from each concentration was mixed with 200 *µ*L of 0.2 mM DPPH solution in 1% methanol. The suspension left incubated for half an hour in dark condition, and the absorbance was measured at 517 nm using a UV double beam spectra scan. The blank contained the same amount of DPPH solution. The absorbance of all samples was recorded at every 5 minutes interval. This experiment was carried out in triplicates, and the antioxidant activity of extracts was calculated by using the following formula:

(2) $$\begin{array}{c} \mathrm{free}\;\mathrm{radical}\;\mathrm{scavenging}\;\mathrm{activity}\left(\%\right)=\frac{\mathrm{absorbance}\;\mathrm{of}\;\mathrm{control}-\mathrm{absorbance}\;\mathrm{of}\;\mathrm{sample}}{\mathrm{absorbance}\;\mathrm{of}\;\mathrm{control}}\times 100. \end{array}$$

### 2.6. Experimental Animals

72 male Wistar albino rats aged between 8 and 9 weeks have been purchased from the Pharmacy Department of Jahangirnagar University (Dhaka, Bangladesh). The animals weighed between 110 gm and 120 gm and were sorted into 12 groups in separate polypropylene cages where each cage contained 6 rats. They were accommodated under temperature (25 ± 2°C) and humidity-controlled zone to maintain an adequate light-dark cycle (12 : 12 hours). There was a consistent supply of water and diet ad libitum to all animals, and they were allowed to acclimatize for ten days before the experiment. Rats of each group underwent euthanasia during all surgical procedures, and anesthesia was conducted by supplying isoflurane (5% in 100% oxygen). Humanitarian care was taken throughout the experiments as per the guidelines demonstrated in the Guide for the Care and Use of Laboratory Animals (NIH publication No: 85-23, revised in 1985). All protocols were authorized by the Biomedical Research Center, University of Dhaka, Bangladesh (Reference No: BMRC/EC/2016-17/98) before commencing the experiments.

### 2.7. Development of the Atherogenic Model

The nutritional composition is vitally recognized to be associated with dysmetabolic diseases like obesity, diabetes, and cardiovascular diseases [37]. Moreover, consumption of a high-saturated fat diet by humans results in an immense risk of developing diabetes and heart disease [38–40]. Application of a high-fat diet (HFD), consisting of saturated fat, and cholesterol, in the rodent model, reported elevating lipid levels [41, 42]. According to the earlier pathological evidence, hyperlipidemia is the chief precondition for the development of atherosclerosis leading to cardiovascular disability and death [43]. The present experiment recruited a previously established high-fat diet composition with a slight modification to develop the atherogenic rat model (Table 1) [44]. All ingredients of the diet were separately stored at 4°C. Atherosclerosis was induced by feeding HFD to the animals for 12 weeks. Freshly prepared pellets were fed daily. The initial and final body weight of all animals were recorded by digital weighing balance. Standard laboratory animal feed for the normal control group of animals has been purchased from the Pharmacy Department of Jahangirnagar University (Dhaka, Bangladesh), which consisted of 5% ash, 23% crude protein, 10% crude fibre, 7% fat, 50% carbohydrate, 0.6% vitamins, and minerals and the remaining is water (4.4%). The atherogenic rat model development was assessed by estimating the change in body weight, total serum lipid content, systolic blood pressure, atherogenic index, and analyzing histopathology of rat aorta.

### 2.8. Treatment Protocol

After 12 weeks of consumption of HFD, different doses of methanolic and ethanolic extracts of *A. ilicifolius* leaf and stem were administered to the animals for 15 consecutive days [45]. All extracts and standard drugs (Simvastatin and Warfarin) were diluted in 2% Tween 80 and administered orally (5 mL/kg of body weight, p.o.) to animals with the help of oral intubation. In order to evaluate the anti-atherosclerotic/anti-atherogenic, antioxidant, anti-hypertensive, and anticoagulative properties of the plant extracts, standard doses of alcoholic extracts of the leaf (250 and 500 mg/kg, b.w.) [46] and stem (200 mg/kg of b.w. and 400 mg/kg of b.w.) [47] have been selected based on the previous report with a little modification and also from the observation of acute toxicity study on animals using different doses (as mentioned below). Selected doses were dispensed to different groups of experimental rats. Rats were allocated into 12 groups and subsequently received treatments, as shown in Table 2.

### 2.9. Acute Toxicity Study

Acute oral toxicity study was performed to ensure the safety of selected doses, based upon the previous report with slight modification [45], by following a standard protocol stated in the organization of economic cooperation and development (OECD) guidelines 423. Total 15 healthy rats were allocated into 5 groups, where each group contained 3 rats. Animals were weighed and fasted overnight prior to the administration of various plant extracts at doses of 300, 400, 500 600, and 700 mg/kg b.w., p.o. and observed at every 30 minutes interval for 24 hours of each dosing for 14 days. Any physical signs of toxicity like pain, palpitation, reduced respiration, or mortality were noted for each animal.

### 2.10. Estimation of Systolic Blood Pressure

After 15 days of treatment, systolic blood pressure of conscious rats was measured in a 30°C environment by the tail-cuff method using BP Monitor Mk-1030, Mouromachi Kikaj Co. Ltd., at the Phytochemical Laboratory of Dhaka University, Bangladesh. An average of three measurements per rat was recorded. Animals were allowed to acclimatize for normal blood circulation in the tail by placing them in the holder for 10 to 15 minutes before taking readings [48].

### 2.11. Blood Sample Collection

At the end of the experiment, 5 mL of blood samples of rats from each group was collected after an overnight fast by retro-orbital puncture under general anesthesia. Centrifugation was done at 3000 rpm for 10 minutes at 4°C in order to separate serum, and the samples were preserved at −80°C for future analysis.

### 2.12. Analysis of Lipid Profile

The serum levels of total cholesterol (TC), triglyceride (TG), and high-density lipoprotein cholesterol (HDL-C) were determined by using previously established protocols [49–51]. The Friedewald formula [52] has been used to calculate serum low-density lipoprotein cholesterol (LDL-C = TC–HDL-C–TG/5). The atherogenic index was estimated using the equation given below [53]: atherogenic index = Log10 (TG/HDL-C).

### 2.13. Determination of Anticoagulant Activity

The plant extracts' anticoagulant properties were performed to further add value to the usage of *A. ilicifolius* as a potential source of the herb against heart diseases since synthetic blood thinners like Warfarin are often prescribed for the treatment of atherosclerosis.

### 2.14. APTT and PT Assays Ex Vivo

The *ex vivo* assays were carried out after treatment with crude extracts for 15 days. For animals from each group (*n* = 6), including normal control and standard, blood was collected after 120 minutes of the last serving dose [54]. Both APTT (activated partial thromboplastin time) and PT (prothrombin time) assays were performed by using commercially available reagent kits and following previously established methods using Warfarin (2 mg/kg body weight, p.o.) as a standard drug [55, 56].

### 2.15. In Vivo Clotting Time Assay

Blood samples of rats were subjected to measurement of whole blood clotting time after the treatment using the capillary glass tube method [57]. Samples were collected 90 minutes after the last dose via retro-orbital plexus using a capillary glass tube, which was placed on a glass-slide to permit coagulation [55]. Following this, a dry needle was used to agitate the blood every 30 seconds, and this was continued until the needle wire aroused a fibrous protein representing the clotting time [58].

### 2.16. In Vivo Bleeding Time Assay

*In vivo* assay of bleeding time was estimated in rats after 90 minutes of administration of the final dose. Each rat's tail was marked and cut at 5 mm long and subsequently submerged in saline water at 37°C. An intervening time between the cutting tail tip and bleeding was recorded as bleeding time [59].

### 2.17. Histopathology

Rat aorta, liver, and kidney were subjected to histopathological examinations at the end of the experiment. These organs were excised and submerged into freshly prepared fixative solution (10% formalin) for 48 hours at 37°C. Cross-sections of paraffin-embedded aorta, liver, and kidney with 5 *µ*m thickness were exposed to hematoxylin and eosin staining. The tissue assessment was executed in Exim Bank Hospital, Department of histopathology, Dhaka, Bangladesh.

### 2.18. Statistical Analysis

Data of the present work were represented as mean ± SEM (standard error mean) (*n* = 6). The experimental values of treatment and normal control groups were compared against the atherogenic model by applying one-way analysis of variance (ANOVA) subsequent to Dunnett's test where *p* < 0 .05 was expressed as a 95% level of statistical significance. Graphpad Instat version 3.10 was used for statistical analysis.

## 3. Results

### 3.1. Qualitative Phytochemical Analysis

The preliminary phytochemical screening process revealed that both leaf and stem of the plant extracts contain significant phytochemical constituents, including alkaloids, flavonoids, phenol, glycosides, steroids, terpenoids, and saponins, as represented in Table 3.

### 3.2. Analysis of the Antioxidant Activity of Plant

The DPPH assay determined the percentage of antiradical efficiency of leaf and stem extracts of *A. ilicifolius* as summarized in Table 4. The IC50 values of the assay are presented in Table 5. The data were compared with standard ascorbic acid. An exponential increase in antioxidant activities with increased concentration of the plant extracts was observed. Maximum inhibition of DPPH free radical was found in AILM (76.45 ± 0.24% at 100 *µ*g/mL), showing the lowest IC_50_ value (5.89 *µ*g/mL). Significant differences were noted (*p* < 0.01) in case of both leaf and stem extracts when compared with ascorbic acid. However, AISE exhibited a result (62.30 ± 0.43%) that corresponds to ascorbic acid data at 10 *µ*g/mL.

### 3.3. Acute Toxicity Test

After administering different doses (300, 400, 500, 600, and 700 mg/kg b.w., p.o.) of plant extracts for two weeks, no behavioral abnormality, physical change, or mortality was observed in rats. Moreover, no toxic reaction was observed at any given dose until the end of the experiment. As a result, the doses 250 and 500 mg/kg b.w./day (p.o.) of alcoholic extracts of leaf and 200 and 400 mg/kg b.w./day (p.o.) of the stem of *A. ilicifolius* were selected as safe doses to carry out further pharmacological investigations for the present work.

### 3.4. Changes in Body Weight

The mean body weight (*n* = 6) of rats reduced after treatment with the extracts for 15 days. The changes in mean body weight of rats have been represented in percentage as given in Table 6. Around 65% of body weight was gained by the animals that consumed a high-fat diet for 12 weeks. The maximum percentage of bodyweight reduction was observed in rats administered 250 mg/kg b.w., p.o. (13.33%) of AILM and 400 mg/kg bw, p.o. (12.17%) of AISM at the end of week 14 (Table 6).

### 3.5. Evaluation of Systolic Blood Pressure

**Figure 1:**
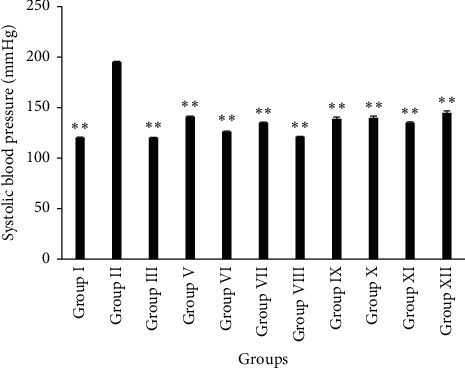
Estimation of systolic blood pressure of rats at the end of the treatment. Results were expressed in mean ± SEM (*n* = 6). (^*∗*^) indicates statistically significant difference from HFD group using ANOVA, followed by Dunnett's multiple comparison test (^*∗*^*p* < 0.05, ^*∗∗*^*p* < 0.01) where treatment groups were compared with Group II (atherogenic control). Group I = normal control, Group II = Atherogenic control (HFD), Group III = atherogenic control + 3 mg/kg of Simvastatin, Group V = atherogenic control + 250 mg/kg ethanolic extract of *A. ilicifolius* (leaf), Group VI = atherogenic control + 500 mg/kg ethanolic extract of *A. ilicifolius* (leaf), Group VII = atherogenic control + 250 mg/kg methanolic extract of *A. ilicifolius* (leaf), Group VIII = atherogenic control + 500 mg/kg methanolic extract of *A. ilicifolius* (leaf), Group IX = atherogenic control + 200 mg/kg methanolic extract of *A. ilicifolius* (stem), Group X = atherogenic control + 400 mg/kg methanolic extract of *A. ilicifolius* (stem), Group XI = atherogenic control + 200 mg/kg ethanolic extract of *A. ilicifolius* (stem), Group XII = atherogenic control + 400 mg/kg ethanolic extract of *A. ilicifolius* (stem).

Treatment with alcoholic extracts of *A. ilicifolius* (leaf and stem) after 15 days significantly decreased (*p* < 0.01) systolic blood pressure of rats when compared with the atherogenic model (Group II) (Figure 1). Consumption of high-fat diet by the experimental animals caused elevated blood pressure (195.5 ± 0.46 mmHg). The systolic blood pressure reduced maximum when treated with 500 mg/kg b.w. (p.o.) of AILM (121.25 ± 0.32 mmHg) that corresponded to normal level (120.5 ± 0.46 mmHg). Administration of Simvastatin (3 mg/kg b.w., p.o.) also reduced the systolic blood pressure in animals (120.25 ± 0.32 mmHg) to the normal level.

### 3.6. Assessment of Lipid Profile

**Figure 2:**
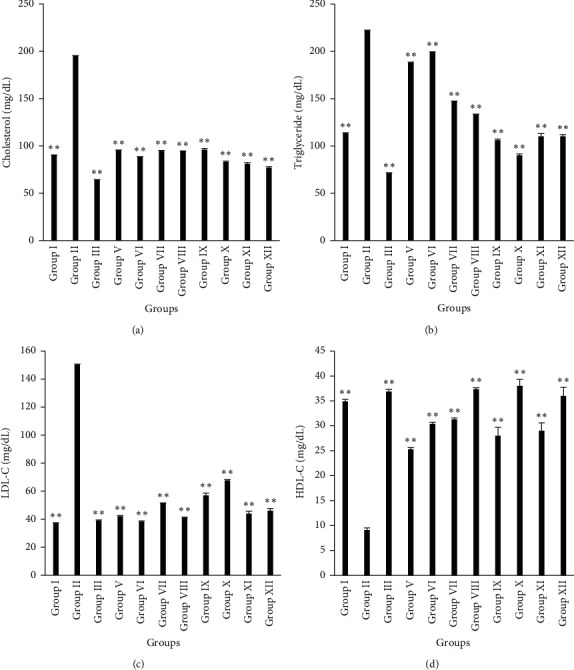
Estimation of cholesterol (a), triglyceride (b), LDL-C (c), HDL-C (d) in rats at the end of the treatment. Results are expressed in mean ± SEM (*n* = 6). (^*∗*^) indicates statistically significant difference from HFD group using ANOVA, followed by Dunnett's multiple comparison test (^*∗*^*p* < 0.05, ^*∗∗*^*p* < 0.01) where treatment groups were compared with Group II (atherogenic control). Group I = normal control, Group II = atherogenic control (HFD), Group III = atherogenic control + 3 mg/kg of Simvastatin, Group V = atherogenic control + 250 mg/kg ethanolic extract of *A. ilicifolius* (leaf), Group VI = atherogenic control + 500 mg/kg ethanolic extract of *A. ilicifolius* (leaf), Group VII = atherogenic control + 250 mg/kg methanolic extract of *A. ilicifolius* (leaf), Group VIII = atherogenic control + 500 mg/kg methanolic extract of *A. ilicifolius* (leaf), Group IX = atherogenic control + 200 mg/kg methanolic extract of *A. ilicifolius* (stem), Group X = atherogenic control + 400 mg/kg methanolic extract of *A. ilicifolius* (stem), Group XI = atherogenic control + 200 mg/kg ethanolic extract of *A. ilicifolius* (stem), Group XII = atherogenic control + 400 mg/kg ethanolic extract of *A. ilicifolius* (stem). LDL-C = low-density lipoprotein cholesterol; HDL-C = high-density lipoprotein cholesterol.

The effect of the plant extracts on the lipid profile of the experimental animals is demonstrated in Figure 2. The levels of serum TC, TG, and LDL-C significantly reduced (*p* < 0.01) while HDL-C significantly escalated (*p* < 0.01) at the end of treatment with alcoholic leaf and stem extracts of *A. ilicifolius* when compared against the atherogenic control group. The serum TC, TG, and LDL-C markedly elevated in high-fat diet-fed rats (194.95 ± 0.39 mg/dL; 222.15 ± 0.43 mg/dL and 150.33 ± 0.32). In contrast, a substantial fall in serum HDL-C level was found in consuming high-fat diet (9.10 ± 0.42 mg/dL). Administration of 400 mg/kg b.w./day (p.o.) of AISE and AISM extensively lowered serum cholesterol and triglyceride respectively (77.00 ± 1.08 mg/dL and 90.00 ± 1.47 mg/dL). Conversely, treatment with 400 mg/kg b.w./day (p.o.) of AISM exhibited maximum upraise in serum HDL-C level (38.00 ± 1.29 mg/dL).

Estimation of the atherogenic index and serum ratios of TC/HDL-C and LDL-C/HDL-C of different groups of rats are summarized in Table 7. A significant difference (*p* < 0.05 and *p* < 0.01) in the atherogenic index of different treatment groups was compared with the atherogenic animals. However, oral administration of 400 mg/kg b.w./day of AISM for 15 days strikingly reduced atherogenic index (0.36 ± 0.12) that is relevant to the data of standard drug (3 mg/kg b.w./day, p.o. of Simvastatin) (0.28 ± 0.10). In comparison to the atherogenic model, the ratios of TC/HDL-C and LDL-C/HDL-C were significantly lower (*p* < 0.01) in normal control and treatment groups. Among the treatment groups, Group XII and Group VIII demonstrated the highest depletion of serum ratio of TC/HDL-C and LDL-C/HDL-C, respectively.

### 3.7. The Anticoagulant Effect of *A. ilicifolius*

**Figure 3:**
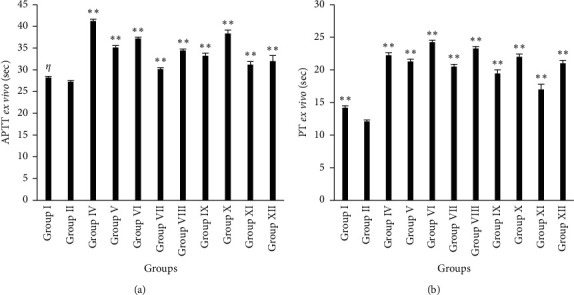
Anticoagulant effect of *A. ilicifolius* in rats; activated partial thromboplastin time assay (a) and prothrombin time assay (b). Results are expressed in mean ± SEM (*n* = 6). (^*∗*^) indicates statistically significant and (*η*) indicates non-significant difference from HFD group using ANOVA, followed by Dunnett's multiple comparison test (^*∗*^*p* < 0.05, ^*∗∗*^*p* < 0.01) where treatment groups were compared with Group II (atherogenic control). Group I = normal control, Group II = atherogenic control (HFD), Group IV = atherogenic control + Warfarin 2 mg/kg, Group V = atherogenic control + 250 mg/kg ethanolic extract of *A. ilicifolius* (leaf), Group VI = atherogenic control + 500 mg/kg ethanolic extract of *A. ilicifolius* (leaf), Group VII = atherogenic control + 250 mg/kg methanolic extract of *A. ilicifolius* (leaf), Group VIII = atherogenic control + 500 mg/kg methanolic extract of *A. ilicifolius* (leaf), Group IX = atherogenic control + 200 mg/kg methanolic extract of *A. ilicifolius* (stem), Group X = atherogenic control + 400 mg/kg methanolic extract of *A. ilicifolius* (stem), Group XI = atherogenic control + 200 mg/kg ethanolic extract of *A. ilicifolius* (stem), Group XII = atherogenic control + 400 mg/kg ethanolic extract of *A. ilicifolius* (stem).

The results of *ex vivo* coagulation assays for APTT and PT are demonstrated in Figure 3. The clotting time of APTT and PT was observed to be significantly high (*p* < 0.01) in rats treated with various plant extracts compared with atherogenic control after completion of dosing. Group X showed a maximum increase in APTT clotting time (38.35 ± 0.83 sec), which was similar to the standard drug (2 mg/kg b.w./day, p.o. of Warfarin) (41.23 ± 0.44 sec). Animals of Group VI showed a larger clotting time of PT (24.23 ± 0.32 sec) than the group that received treatment with a standard drug (22.23 ± 0.41 sec). However, there were no significant changes in APTT clotting time found in rats of the normal control group (28.23 ± 0.30 sec) as compared to the atherogenic control group (27.23 ± 0.34 sec).

**Figure 4:**
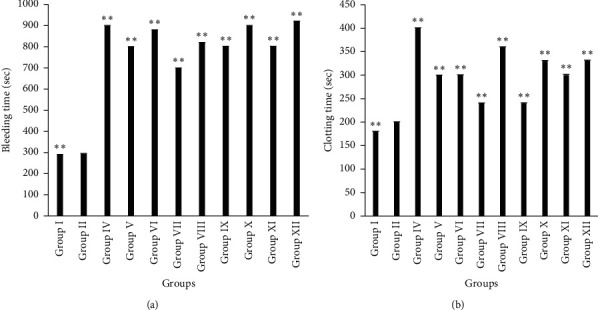
Determination of bleeding (a) and clotting (b) time in rats. Results are expressed in mean ± SEM (*n* = 6). (^*∗*^) indicates statistically significant difference from HFD group using ANOVA, followed by Dunnett's multiple comparison test (^*∗*^*p* < 0.05, ^*∗∗*^*p* < 0.01) where treatment groups were compared with Group II (atherogenic control). Group I = normal control, Group II = atherogenic control (HFD), Group IV = atherogenic control + Warfarin 2 mg/kg, Group V = atherogenic control + 250 mg/kg ethanolic extract of *A. ilicifolius* (leaf), Group VI = atherogenic control + 500 mg/kg ethanolic extract of *A. ilicifolius* (leaf), Group VII = atherogenic control + 250 mg/kg methanolic extract of *A. ilicifolius* (leaf), Group VIII = atherogenic control + 500 mg/kg methanolic extract of *A. ilicifolius* (leaf), Group IX = atherogenic control + 200 mg/kg methanolic extract of *A. ilicifolius* (stem), Group X = atherogenic control + 400 mg/kg methanolic extract of *A. ilicifolius* (stem), Group XI = atherogenic control + 200 mg/kg ethanolic extract of *A. ilicifolius* (stem), Group XII = atherogenic control + 400 mg/kg ethanolic extract of *A. ilicifolius* (stem).

Furthermore, the results of *in vivo* bleeding time assay revealed that high doses of plant extracts (Group VI, Group X, and Group XII) showed data (880.20 ± 0.33; 900.00 ± 0.82, and 920 ± 1.6 sec, respectively) similar to Warfarin (900.00 ± 0.44 sec) as demonstrated in Figure 4. Nevertheless, clotting time was markedly prolonged in rats of Group VIII (359.98 ± 0.43 sec).

## 4. Effect of the Alcoholic Extracts of *A. ilicifolius* on Histopathology of Aorta, Liver, and Kidney

### 4.1. Aorta

**Figure 5:**
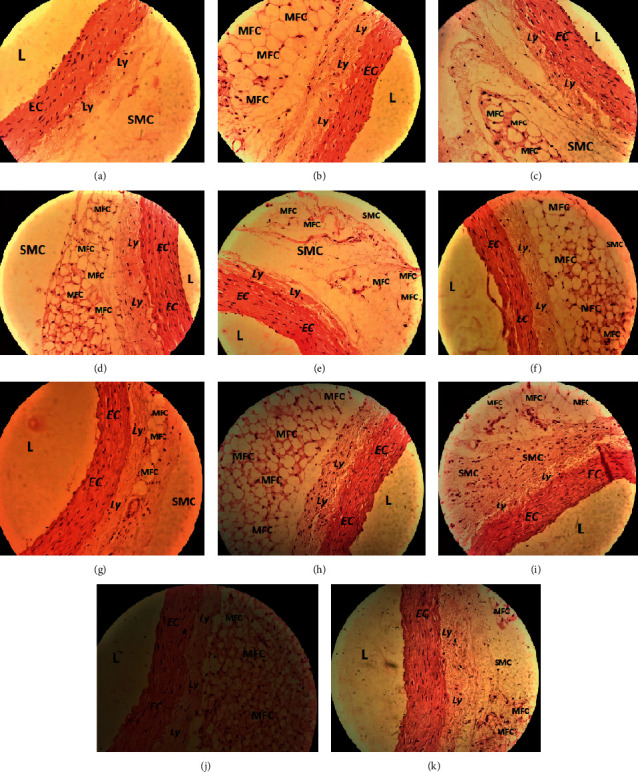
Histopathology of rat aorta showing vascular lumen (L), endothelial cells (EC), group of lymphocytes (Ly), smooth muscle cells (SMC), and macrophage-derived foam cells (MFC). (a): Group I = normal control, (b): Group II = atherogenic control (HFD), (c): Group III = atherogenic control + 3 mg/kg of Simvastatin, (d): Group V = atherogenic control + 250 mg/kg ethanolic extract of *A. ilicifolius* (leaf), (e): Group VI = atherogenic control + 500 mg/kg ethanolic extract of *A. ilicifolius* (leaf), (f): Group VII = atherogenic control + 250 mg/kg methanolic extract of *A. ilicifolius* (leaf), (g): Group VIII = atherogenic control + 500 mg/kg methanolic extract of *A. ilicifolius* (leaf), (h): Group IX = atherogenic control + 200 mg/kg methanolic extract of *A. ilicifolius* (stem), (i): Group X = atherogenic control + 400 mg/kg methanolic extract of *A. ilicifolius* (stem), (j): Group XI = atherogenic control + 200 mg/kg ethanolic extract of *A. ilicifolius* (stem), (k): Group XII = atherogenic control + 400 mg/kg ethanolic extract of *A. ilicifolius* (stem). Microscopic examination of each group was performed at 400X magnification, scale bar: 40 *μ*m.

Histopathological changes of the cross section of rat aorta after treatment with different doses of plant extracts are demonstrated in Figure 5. Under the microscopic examination, a normal cardiac aorta architecture was observed in rats of a normal control group where the endothelial cells and smooth muscle cells are underlying much prominently (Figure 5(a)). Conversely, atherogenic rat aorta showed excessive accumulation of lipid droplets had been found causing the development of innumerable macrophage-derived foam cells (MFC) under the thin endothelium lining, which resulted in the migration of smooth muscle cell (SMC) within the area of lipid droplets resulting formation of atherosclerotic lesions with substantial destruction of smooth muscle cells, endothelial cells, and lymphocytes cells (Figure 5(b)). Administered high doses (500 and 400 mg/kg b.w./day, p.o., respectively) of AILM, AILE, and AISE exhibited aorta with fewer macrophage-derived foam cells and lesser destruction of endothelial cells when compared to the atherogenic control group (Figures 5(g), 5(i), and 5(k)). A similar observation was made in rats treated with standard drugs showing much conserved cellular architecture (Figure 5(c)).

### 4.2. Liver

**Figure 6:**
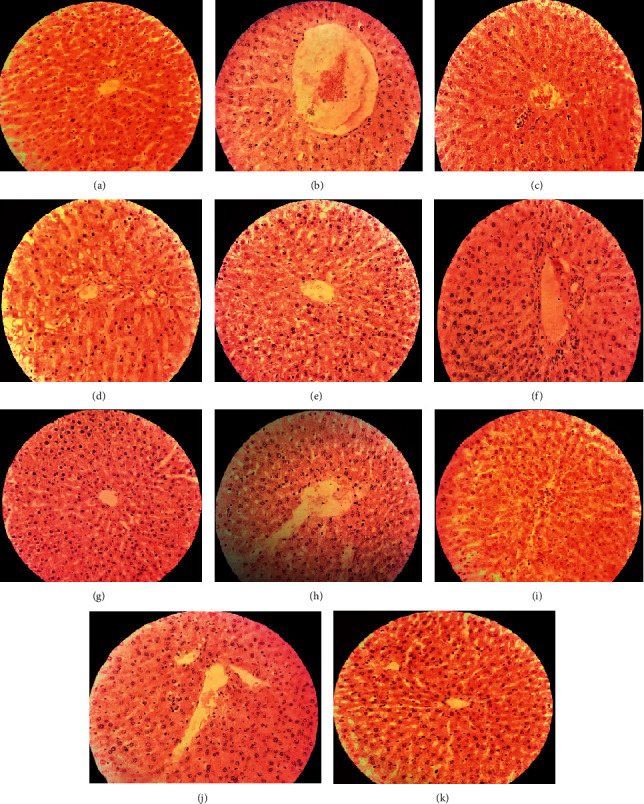
Histopathology of rat liver; (a): Group I = normal control, (b): Group II = atherogenic control (HFD), (c): Group III = atherogenic control + 3 mg/kg of Simvastatin, (d): Group V = atherogenic control + 250 mg/kg ethanolic extract of *A. ilicifolius* (leaf), (e): Group VI = atherogenic control + 500 mg/kg ethanolic extract of *A. ilicifolius* (leaf), (f): Group VII = atherogenic control + 250 mg/kg methanolic extract of *A. ilicifolius* (leaf), (g): Group VIII = atherogenic control + 500 mg/kg methanolic extract of *A. ilicifolius* (leaf), (h): Group IX = atherogenic control + 200 mg/kg methanolic extract of *A. ilicifolius* (stem), (i): Group X = atherogenic control + 400 mg/kg methanolic extract of *A. ilicifolius* (stem), (j): Group XI = atherogenic control + 200 mg/kg ethanolic extract of *A. ilicifolius* (stem), (k): Group XII = atherogenic control + 400 mg/kg ethanolic extract of *A. ilicifolius* (stem). Microscopic examination of each group was performed at 400X magnification, scale bar: 40 *μ*m.

The liver histology of rats varied among different groups, as represented in Figure 6. Normal histology of liver tissue was observed in Group I (Figure 6(a)). However, extensive fat deposition around the central vein area was seen in the microscopic image of liver tissue of rats that consumed high-fat diet resulting in an enlarged central vein, infiltration of inflammatory cells with cellular necrosis, and development of fibrosis (Figure 6(b)). This confirmed the evolution of microvesicular steatosis in the liver. The oral medication of 500 mg/kg b.w./day of AILM and 400 mg/kg b.w./day of AISM and AISE showed much-improved liver tissues with the presence of compact and healthy hepatocytes (Figures 6(g), 6(i), and 6(k)). This was the case found in rats medicated with 3 mg/kg b.w./day, p.o. of Simvastatin (Figure 6(c)).

### 4.3. Kidney

**Figure 7:**
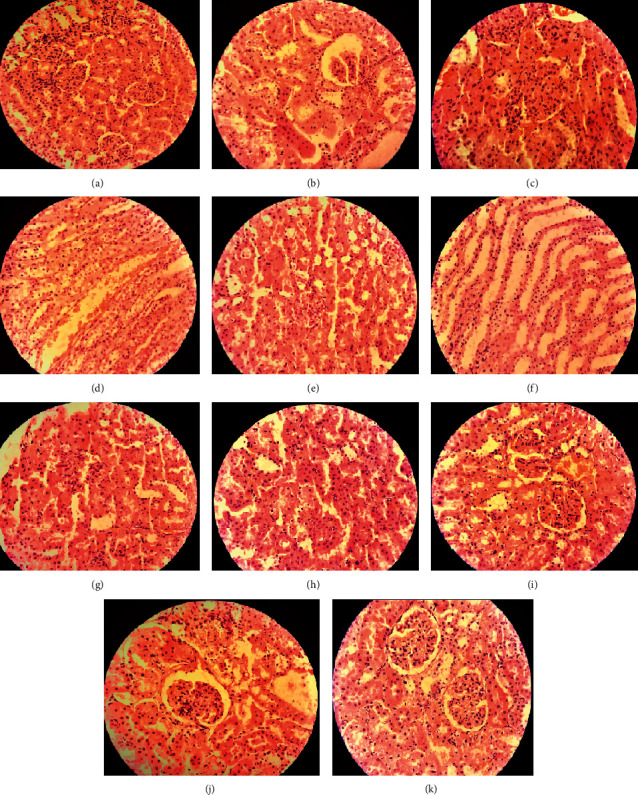
Histology of rat kidney; (a): Group I = normal control, (b): Group II = atherogenic control (HFD), (c): Group III = atherogenic control + 3 mg/kg of Simvastatin, (d): Group V = atherogenic control + 250 mg/kg ethanolic extract of *A. ilicifolius* (leaf), (e): Group VI = atherogenic control + 500 mg/kg ethanolic extract of *A. ilicifolius* (leaf), (f): Group VII = atherogenic control + 250 mg/kg methanolic extract of *A. ilicifolius* (leaf), (g): Group VIII = atherogenic control + 500 mg/kg methanolic extract of *A. ilicifolius* (leaf), (h): Group IX = atherogenic control + 200 mg/kg methanolic extract of *A. ilicifolius* (stem), (i): Group X = atherogenic control + 400 mg/kg methanolic extract of *A. ilicifolius* (stem), (j): Group XI = atherogenic control + 200 mg/kg ethanolic extract of *A. ilicifolius* (stem), (k): Group XII = atherogenic control + 400 mg/kg ethanolic extract of *A. ilicifolius* (stem). Microscopic examination of each group was performed at 400X magnification, scale bar: 40 *μ*m.

The morphology of normal rat kidneys showed a regular arrangement of cells in the outer portion of the medulla of the kidney with minimum lipid deposition and many prominent glomeruli (Figure 7(a)). However, abnormal histology of rat kidneys was seen in groups that consumed lipid-rich diet and exhibited the presence of considerable fat accumulation around glomeruli and infiltration of inflammatory cells (Figure 7(b)). Pathological destruction of the kidney tissues was substantially reduced and normal architecture of glomeruli, collecting ducts, tubules, ascending, and descending loops of the kidney were observed in animals treated with the standard drug (Figure 7(c)) as well as those that received 400 mg/kg b.w./day, p.o. of AISE and AISM (Figures 7(i) and 7(k)).

## 5. Discussion

*A. ilicifolius* is acknowledged for its rich sources of several phytochemical constituents like steroids, triterpenoids, saponins, flavonoids, alkaloids, and tannins [3]. The whole and distinct plant parts have been employed for the purpose of therapeutic aid [60, 61]. In our present study, alcoholic extracts of *A. ilicifolius* leaf and stem have been recruited to investigate its potentiality against atherosclerosis in the high-fat diet-fed rat model. We found several classes of bioactive compounds in both stem and leaf extracts of the plant (Table 3) that possess many health benefits due to their effectiveness in the process of drug discovery in traditional and pharmaceutical industries [62]. Literature data revealed that phenolic compounds are abundantly present in the alcoholic extracts of different parts of *A. ilicifolius*, particularly root and leaf [63–66]. These data support that the plant extract are directly associated with antioxidant activities since their hydroxyl group potentially absorbs and detoxifies the free radicals [67–72]. Free radicals are known to produce oxidative stress, for which several conditions like diabetes, atherosclerosis, arthritis, and seizure may arise [73]. This can be avoided by the consumption of antioxidants from natural sources that create a balance between the availability of free radicals and endogenous antioxidants in blood [74, 75]. The current study displayed the antioxidant power of the plant extracts even at the lowest concentration (1.25 *µ*g/mL), indicating a considerable amount of phenolic compounds within the extract. The study further revealed that the leaf extracts of *A. ilicifolius* (AILM and AILE) possess better antioxidant properties with the lowest IC_50_ values (5.89 *µ*g/mL and 6.59 *µ*g/mL) as compared to the root extracts of the plant. Besides, the leaves of *A. ilicifolius* were found as a potential source of natural antioxidants in a study of the recent year [72]. Nevertheless, the antioxidant capacity of plant extracts was found less than that of ascorbic acid, which reflects the results from previous studies on this mangrove plant material [19, 76].

Regular consumption of high-fat diet (HFD) leads to obesity, which encourages the body to store more fat within the tissues and organs and gain weight [40]. The presence of a prominent amount of fat in diet also gives rise to aortic atherosclerosis by elevating serum TC, TG, and LDL-C [76]. Our study demonstrated that the administration of plant extracts potentially reduced body weight. Obesity associated with dyslipidemia is considered one of the prominent causes of the evolution of cardiovascular diseases [77]. A high concentration of cholesterol in the blood leads to oxidized LDL-C, which is engulfed by existing macrophages within endocardial endothelium as lipid-rich foam cells, and eventually develops lesions of atherosclerotic plaque inside the arterial wall [77]. Hence, minimization of serum cholesterol, triglyceride, and LDL-C levels plays a prominent role in the deterrence of cardiovascular disorders, which could be achieved by administering antioxidants from natural sources [77, 78].

According to the present experiment, analysis of total lipid concentrations manifested that 400 mg/kg b.w./day, p.o. of AISM extensively declined serum triglyceride and cholesterol levels in rats. In comparison, maximal depletion of total LDL-C level was found in rats receiving medication of 500 mg/kg b.w./day, p.o. of AILE. Nevertheless, the highest serum HDL-C level was observed in animals that received 400 mg/kg b.w./day, p.o. of AISM. A low HDL-C level and a high level of LDL-C level are directly linked to the likelihood of various cardiovascular diseases since HDL-C potentially transfers excess cholesterol from the arteries and tissues [79, 80].

An elevated atherogenic index is considered one of the significant markers of atherosclerosis [11]. In our study, the atherogenic condition promisingly improved in animals that encountered a dose of 400 mg/kg b.w./day, p.o. of AISE and AISM. Several past pieces of evidence underpin the fact that a lipid-rich diet is potentially accountable for vascular damage and oxidative stress due to the presence of excessive TC, TG, and LDL-C in the blood, which could lead to clinical consequences like heart attack, stroke, or other fatal condition [81, 82]. Furthermore, unconventional fat metabolism in the body helps expand the ratios of TC/HDL-C and LDL-C/HDL-C in serum [83]. The present observation evidenced that high doses (400 and 500 mg/kg b.w./day, p.o.) of AILM and AISM strongly declined these ratios confirming significant lowering of fat content within the body. The presence of phenols and flavonoids can justify this statement and terpenoids in the plant extract. They strive for the antioxidant effect that controls lipid metabolism by minimizing inflammatory, fibro-proliferative, and vascular disorders [84–88].

The histopathology report proclaimed the atherosclerotic lesion incident within the arterial walls of high-fat diet-fed rats improved by high doses (400 and 500 mg/kg b.w./day, p.o.) of the plant extracts. Besides, a stockpile of fat within the tissues massively interrupted the regular morphology of liver and kidney tissues of atherogenic rats that were ameliorated to near normalcy by high doses of plant extracts.

The diuretic properties of *A. ilicifolius* were evidenced by a previous study where the presence of flavonoids and glycosides was believed to produce diuresis [89]. Regarding the fact that diuretics are used as therapy for hypertension, the present investigation was deliberated to assess the anti-hypertensive properties of *A. ilicifolius*. Current data showed treatment with 500 mg/kg b.w./day (p.o.) of AILM potentially lowered SBP to a normal level. Literature data support the certainty of the relationship between oxidative stress and hypertension progression by building up uncontrolled free radicals like ROS (reactive oxygen species) [90–92]. Moreover, mangrove species like *A. ilicifolius* consist of many antioxidant enzymes that exhibit defensive action against the detrimental consequences of ROS [93]. On that account, our work suggests that the antioxidant response by different extracts of *A. ilicifolius* could be the reason for their anti-hypertensive action. Besides, former research reported that a high HDL-C level contributes to the de-escalation of blood pressure [94], which corresponds to our present results.

Furthermore, the ethanolic leaf and aqueous root extracts of *A. ilicifolius* have been reported to possess anticoagulant potentiality at a higher dose in recent studies [72, 94]. Our study manifested that high doses (400 and 500 mg/kg b.w./day, p.o.) of AILM and AISE predominantly decreased blood coagulation time. The accountability for the attainment of anticoagulant action of the plant extracts could be due to high level of flavonoids since compounds that exert antioxidant effect are responsible for anticoagulation [95].

Our work demonstrated that alcoholic extracts of both leaf and stem of *A. ilicifolius* could be a potential source of multiple phytoconstituents that play essential roles in forestalling oxidative stress, atherosclerosis, hypertension, and blood clotting within the body. In general, alcohols, explicitly methanol, are considered promising solvent systems for extraction technique since a number of active compounds, namely, flavonoids, phenols, tannins, and saponins, are found on phytochemical screening of plant extracts [96]. Methanol, however, turned up to be a better solvent system based on previous findings as the solvent is more effective in dissolving the active chemical moieties in the cells as it helps to perforate cellular membrane of the plant material to infiltrate the intracellular components [19].

## 6. Conclusion

To summarize, the present investigation documented both leaf and stem of *A. ilicifolius* as beneficiary herbs for the prevention of cardiac diseases by ameliorating various conditions like oxidative stress, obesity, hyperlipidemia, and high blood pressure. Based on biochemical tests and histopathology of distinguished tissues (aorta, liver, and kidney), our study manifested that alcoholic leaf and stem extracts were effective against atherosclerosis in a dose-dependent approach. Also, the blood-thinning properties of the plant extract further support its usefulness as a medicinal plant for the management of cardiovascular diseases. The present data confirm that doses of 400 and 500 mg/kg b.w./day (p.o.) of stem and leaf extracts were most effective against all parameters used for assessing the atherogenic condition. However, being a first-hand report, the current work suggests more investigations on quantifying and isolating the active constituents and their metabolites in leaves and stem of *A. ilicifolius* to justify the plant as a promising source of the secondary therapeutic scheme against atherosclerosis.

## Figures and Tables

**Table 1:** Diet (HFD) composition (g/1000 g of diet) (Matos et al. [44]).

Ingredients	Cellulose	Corn starch	Soybean oil	Casein	Choline	Cholesterol	Salts^∗^*∗*	Vitamins^∗∗^*∗∗*
Amount incorporated in the diet (g/kg)	132.00	429.00	260.00	110.00	1.00	15.00	45.00	8.00
Total calories (Kcal)	4538.00							

Salts^∗^*∗* (g/kg of HFD): incorporation of MnSO_4_. H_2_O–4, ZnSO4.7H_2_O– 0.55, FeSO_4_. 7H_2_O–27.3, KH_2_PO_4_–390, CoCl_2_. 6H_2_O–0.03, CuSO_4_. 5H_2_O–0.48, KI–0.80 and NaCl–139 in standard HFD. Vitamins^∗∗^*∗∗* (g/kg of HFD): incorporation of vitamin A-2,100,000 IU, Vitamin D_3_-210,000 IU, PABA-8.00, myo-Inositol-9.00, Vitamin B_3_-3.00, Vitamin B_5_-3.00, Vitamin B_2_-0.90, Vitamin B_1_-0.40, Vitamin B_6_-0.50, Vitamin B_9_-0.20, Vitamin B_7_-0.04, Cyanocobalamin - 0.003, Choline-250.0, Vitamin E-10,000 IU, and Sucrose q.s.p. 1200 in standard HFD.

**Table 2:** Treatment protocol.

Groups	Group I	Group II	Group III	Group IV	Group V	Group VI	Group VII	Group VIII	Group IX	Group X	Group XI	Group XII
Treatment (for 15 days)	Non-atherogenic/normal control rats were fed normal diet + 2% tween 80 (5 mL/kg body weight, p.o.) daily	Atherogenic rats were fed high-fat diet (HFD) + 2% tween 80 (5 mL/kg body weight, p.o.) (atherogenic control) daily	Atherogenic rats were fed HFD + Simvastatin 3 mg/kg body weight, p.o.	Atherogenic rats were fed HFD + Warfarin 2 mg/kg body weight, p.o.	Atherogenic rats were fed HFD + 250 mg/kg b.w./day, p.o. AILE	Atherogenic rats were fed HFD + 500 mg/kg b.w./day, p.o. AILE	Atherogenic rats were fed HFD + 250 mg/kg b.w./day, p.o. AILM	Atherogenic rats were fed HFD + 500 mg/kg b.w./day, p.o. AILM	Atherogenic rats were fed HFD + 200 mg/kg b.w./day, p.o. AISE	Atherogenic rats were fed HFD + 400 mg/kg b.w./day, p.o. AISE	Atherogenic rats were fed HFD + 200 mg/kg b.w./day, p.o. AISM	Atherogenic rats were fed HFD + 400 mg/kg b.w./day, p.o. AISM

AILE = ethanolic extract of *A. ilicifolius* (leaf); AILM = methanolic extract of *A. ilicifolius* (leaf). AISE = ethanolic extract of *A. ilicifolius* (stem); AISM = methanolic extract of *A. ilicifolius* (stem).

**Table 3:** Qualitative phytochemical analysis of the ethanolic and methanolic extracts of *A. ilicifolius* (leaf and stem).

Phytoconstituents	AILE	AILM	AISE	AISM
Alkaloids	+	+	+	+
Flavonoids	+	+	+	+
Phenols	+	+	+	+
Glycosides	+	+	+	+
Steroids	+	+	+	+
Terpenoids	+	+	+	+
Carbohydrates	+	+	+	+
Saponins	+	+	+	+

+ = present, = absent. AILE = ethanolic extract of *A. ilicifolius* (leaf); AILM = methanolic extract of *A. ilicifolius* (leaf). AISE = ethanolic extract of *A. ilicifolius* (stem); AISM = methanolic extract of *A. ilicifolius* (stem).

**Table 4:** DPPH-based antioxidant effect of alcoholic extracts of *A. ilicifolius* (leaf and stem) against standard ascorbic acid.

Concentration (µg/mL)	Mean % inhibition of DPPH free radical				
	Ascorbic acid (standard)	AILM	AILE	AISE	AISM
1.25	44.50 ± 0.41	39.03 ± 0.41^*∗∗*^	26.18 ± 0.44^*∗∗*^	31.60 ± 0.55^*∗∗*^	24.30 ± 0.42^*∗∗*^
2.5	48.25 ± 0.39	40.90 ± 0.37^*∗∗*^	42.01 ± 0.41^*∗∗*^	37.30 ± 0.44^*∗∗*^	28.30 ± 0.40^*∗∗*^
5	51.88 ± 0.18	42.30 ± 0.33^*∗∗*^	51.88 ± 0.43^*∗∗*^	45.60 ± 0.67^*∗∗*^	29.10 ± 0.45^*∗∗*^
10	61.43 ± 0.33	56.20 ± 0.31^*∗∗*^	56.03 ± 0.32^*∗∗*^	62.30 ± 0.43^*η*^	47.10 ± 0.45^*∗∗*^
50	71.50 ± 0.22	73.27 ± 0.24^*∗∗*^	73.19 ± 0.44^*∗∗*^	68.30 ± 0.35^*∗∗*^	66.20 ± 0.48^*∗∗*^
100	91.45 ± 0.26	76.45 ± 0.24^*∗∗*^	74.58 ± 0.39^*∗∗*^	72.60 ± 0.58^*∗∗*^	73.50 ± 0.38^*∗∗*^

AILE = ethanolic extract of *A. ilicifolius* (leaf); AILM = methanolic extract of *A. ilicifolius* (leaf). AISE = ethanolic extract of *A. ilicifolius* (stem); AISM = methanolic extract of *A. ilicifolius* (stem).

**Table 5:** IC_50_ values and regression equations of DPPH assay for different test extracts.

Sample	IC_50_ (*µ*g/mL)	Regression equation	*R* ^2^
AILM	5.89	*y* = 22.141*x* + 32.92	0.956
AILE	6.59	*y* = 24.302*x* + 30.098	0.948
AISE	7.10	*y* = 22.27*x* + 31.051	0.931
AISM	14.80	*y* = 27.921*x* + 17.295	0.963
Ascorbic acid	3.03	*y* = 22.954*x* + 38.93	0.932

AILE = ethanolic extract of *A. ilicifolius* (leaf); AILM = methanolic extract of *A. ilicifolius* (leaf); AISE = ethanolic extract of *A. ilicifolius* (stem); AISM = methanolic extract of *A. ilicifolius* (stem). IC_50_ = inhibitory concentration 50, *y* = dependent variable (% inhibition), i.e., 50%, *x* = independent variable (logarithmic concentration), *R*^2^  = coefficient of determination representing how close the data (dependent and independent variables) are to the fitted regression line; a value close to 100% (1.00) is recognized as best fitted line (linear relationship between dependent and independent variables).

**Table 6:** Measurement of changes in mean body weight of rats (*n* = 6).

Groups	Treatment	Mean body weight (g)			
		Initial body weight	Body weight (week 12)	Body weight (week 14)	Change in body weight after treatment (%)
Group I	Normal diet	120	180	–	–
Group II	High-fat diet (HFD)	115	327	–	–
Group III	Simvastatin 3 mg/kg b.w./p.o.	120	323	298	7.8
Group V	HFD + AILE 250 mg/kg bw/p.o.	110	300	287	4.34
Group VI	HFD + AILE 500 mg/kg b.w./p.o.	110	330	291	11.9
Group VII	HFD + AILM 250 mg/kg b.w./p.o.	110	330	286	13.33
Group VIII	HFD + AILM 500 mg/kg b.w./p.o.	120	312	289	7.38
Group IX	HFD + AISE 200 mg/kg b.w./p.o.	120	320	286	10.63
Group X	HFD + AISE 400 mg/kg b.w./p.o.	110	310	290	6.45
Group XI	HFD + AISM 200 mg/kg b.w./p.o.	110	300	285	5.00
Group XII	HFD + AISM 400 mg/kg b.w./p.o.	115	330	288	12.73

AILE = ethanolic extract of *A. ilicifolius* (leaf), AILM = methanolic extract of *A. ilicifolius* (leaf). AISE = ethanolic extract of *A. ilicifolius* (stem), AISM = methanolic extract of *A. ilicifolius* (stem).

**Table 7:** Estimation of atherogenic index and ratios of serum TC/HDL-C and LDL-C/HDL-C.

Group	Treatment	Atherogenic index	TC/HDL-C	LDL-C/HDL-C
Group I	Normal diet	0.53 ± 0.20^∗^*∗*	2.55 ± 0.03^∗∗^*∗∗*	1.04 ± 0.03^∗∗^*∗∗*
Group II	High-fat diet (HFD)	1.40 ± 0.26	21.49 ± 0.36	16.42 ± 0.36
Group III	Simvastatin 3 mg/kg bw/p.o.	0.28 ± 0.10^∗∗^*∗∗*	1.7 ± 0.23^∗∗^*∗∗*	1.05 ± 0.03^∗∗^*∗∗*
Group V	HFD + AILE 250 mg/kg bw/p.o.	0.88 ± 0.09^*η*^	3.83 ± 0.27^∗∗^*∗∗*	1.8 ± 0.23^∗∗^*∗∗*
Group VI	HFD + AILE 500 mg/kg bw/p.o.	0.68 ± 0.17^*η*^	2.86 ± 0.17^∗∗^*∗∗*	1.34 ± 0.23^∗∗^*∗∗*
Group VII	HFD + AILM 250 mg/kg bw/p.o.	0.64 ± 0.19^∗^*∗*	3.04 ± 0.04^∗∗^*∗∗*	1.57 ± 0.38^∗∗^*∗∗*
Group VIII	HFD + AILM 500 mg/kg bw/p.o.	0.59 ± 0.19^∗^*∗*	2.59 ± 0.26^∗∗^*∗∗*	1.14 ± 0.16^∗∗^*∗∗*
Group IX	HFD + AISE 200 mg/kg bw/p.o.	0.56 ± 0.20^∗^*∗*	3.36 ± 0.24^∗∗^*∗∗*	2.03 ± 0.02^∗∗^*∗∗*
Group X	HFD + AISE 400 mg/kg bw/p.o.	0.36 ± 0.12^∗∗^*∗∗*	2.25 ± 0.19^∗∗^*∗∗*	1.88 ± 0.24^∗∗^*∗∗*
Group XI	HFD + AISM 200 mg/kg bw/p.o.	0.57 ± 0.19^∗^*∗*	2.71 ± 0.35^∗∗^*∗∗*	1.55 ± 0.22^∗∗^*∗∗*
Group XII	HFD + AISM 400 mg/kg bw/p.o.	0.49 ± 0.20^∗∗^*∗∗*	2.18 ± 0.18^∗∗^*∗∗*	1.27 ± 0.30^∗∗^*∗∗*

Values are expressed as mean ± SEM of 6 rats; ANOVA was used for multiple comparison where the treatment groups are compared with Group II. Here, ^*∗*^*p* < 0.05, ^*∗∗*^*p* < 0.01 and *η* = non-significant (*p* > 0.05). TC = total cholesterol; LDL-C = low-density lipoprotein cholesterol; HDL-C = high-density lipoprotein cholesterol; AILE = ethanolic extract of *A. ilicifolius* (leaf), AILM = methanolic extract of *A. ilicifolius* (leaf), AISE = ethanolic extract of *A. ilicifolius* (stem), AISM = methanolic extract of *A. ilicifolius* (stem).

## Data Availability

All data contained within the paper.

## Abbreviations

*A. ilicifolius*: *Acanthus ilicifolius*
DPPH: 2,2-Diphenyl-1-picrylhydrazyl
SBP: Systolic blood pressure
HFD: High-fat diet
TC: Total cholesterol
TG =: Triglyceride
HDL-C: High-density lipoprotein cholesterol
LDL-C: Low-density lipoprotein cholesterol
APTT: Activated partial thromboplastin time
PT: Prothrombin time
SEM: Standard error mean
AILE: Ethanolic extract of *A. ilicifolius* (leaf)
AILM: Methanolic extract of *A. ilicifolius* (leaf)
AISE: Ethanolic extract of *A. ilicifolius* (stem)
AISM: Methanolic extract of *A. ilicifolius* (stem).
